# How are Aging and Osteoarthritis Related?

**DOI:** 10.14336/AD.2022.0831

**Published:** 2023-06-01

**Authors:** Shital Wakale, Xiaoxin Wu, Yogita Sonar, Antonia Sun, Xiwei Fan, Ross Crawford, Indira Prasadam

**Affiliations:** ^1^Centre for Biomedical Technologies, Faculty of Engineering, Queensland University of Technology, Brisbane, Queensland, Australia.; ^2^Orthopaedic Department, The Prince Charles Hospital, Brisbane, Queensland, Australia.

**Keywords:** aging, cartilage, osteoarthritis, oxidative stress, senescence

## Abstract

Osteoarthritis is the most prevalent degenerative joint disease and one of the leading causes of physical impairment in the world's aging population. The human lifespan has significantly increased as a result of scientific and technological advancements. According to estimates, the world's elderly population will increase by 20% by 2050. Aging and age-related changes are discussed in this review in relation to the development of OA. We specifically discussed the cellular and molecular changes that occur in the chondrocytes during aging and how these changes may make synovial joints more susceptible to OA development. These changes include chondrocyte senescence, mitochondrial dysfunction, epigenetic modifications, and decreased growth factor response. The age-associated changes occur not only in the chondrocytes but also in the matrix, subchondral bone, and synovium. This review aims to provide an overview of the interplay between chondrocytes and matrix and how age-related changes affect the normal function of cartilage and contribute to OA development. Understanding the alterations that affect the function of chondrocytes will emerge new possibilities for prospective therapeutic options for the treatment of OA.

## 1. Introduction

Knee Osteoarthritis (OA) is a prevalent chronic degenerative disease that causes severe joint pain and eventually leads to joint dysfunction. Over 500 million people worldwide are affected by OA, and it has a considerable socioeconomic burden on society [1, 2]. Age, genetic predisposition, obesity, inflammation, sports injury, and gender are all risk factors for the development of OA. However, the occurrence of OA increases with advancing age; it is considered the leading risk factor for OA [3]. Even though people of all ages are at risk, the burden of OA falls disproportionately on the elderly. Over 40% of all OA cases occur in those over the age of 65 [4]. Although aging and articular cartilage degeneration in OA are two distinct processes, aging causes changes in the musculoskeletal system that eventually lead to OA development [5, 6]. Several studies have found that as people get older, the frequency and prevalence of synovial joint deterioration increase significantly [7-11]. Moreover, at the age of 50, the occurrence of developing post-traumatic OA following an intraarticular knee fracture increases 3 to 4-fold [12].

The knee joint is the most complex synovial joint composed of multiple structures, including an articular capsule, a subchondral bone, a synovial membrane, and hyaline articular cartilage, which protects the end of bones in a joint. Aging is a multifaceted process that affects the knee joint structure. It leads to changes in the biochemical and biomechanical properties of the knee joint at macroscopic and microscopic levels, affecting its functional performance. These age-related changes include telomere attrition, epigenetic alterations, mitochondrial dysfunction, and, most importantly, changes in the mechanical properties of the cell and changes in the extracellular matrix of the cell [13]. During the process of aging, changes occur at various levels, including cellular changes, changes in the cartilage, osteochondral junction, and also in the synovium, which makes the joint more susceptible to the development of OA [14]. This review provides an overview of the cellular and molecular changes that occur in the synovial joint with age and discusses how age-associated dysregulated cell processes contribute to the development of OA.

## 2. Effects of aging on chondrocytes and its correlation with OA

### 2.1 Chondrocyte senescence and aging

Aging contributes to the accumulation of senescent cells and results in age-related tissue dysfunction. Senescence is a complex process where cells undergo metabolic, morphological, and physiological changes in response to various cellular stressors. Cellular senescence is described by stress-induced cell cycle arrest and the generation of pro-inflammatory paracrine chemicals called senescence-associated secretory phenotype (SASP). Articular cartilage deteriorates with age, and chondrocyte senescence is a crucial factor that plays a very significant role in the development and progression of OA [6, 15-17]. The regenerative ability of mesenchymal stem cells is impaired when senescent chondrocytes are injected into the articular cartilage [18]. However, the in vivo study in the mouse OA model shows a decrease in joint degeneration after the local clearance of senescent cells from articular cartilage [19]. These findings reveal that chondrocyte senescence is a critical part of the development of OA and is important for joint degeneration during aging.

Extrinsic and intrinsic senescence are the two types of cellular senescence. The intrinsic senescence, also known as replicative senescence, is caused by telomere shortening. The stress-induced senescence or extrinsic senescence is caused by different stimuli like activation of oncogenes, oxidative stress, or inflammatory cytokines. Chondrocyte’s senescence is more likely due to stress-induced mechanisms than intrinsic ones [20]. In aged cartilage, senescent chondrocytes exhibit upregulation of matrix-degrading enzymes such as matrix metallo-proteinase 3 (MMP-3) and MMP-13, aggrecanases, as well as the accumulation of damaged collagen. During the aging process, increased expression of MMPs, collagenase, and cathepsin K results in cartilage breakdown [21]. Increased production of these matrix-degrading enzymes damages the cartilage, which leads to the progression of OA.

Oxidative stress is a major cause of stress-induced senescence during the process of aging. An increase in the generation of reactive oxygen species (ROS) or a decrease in the number of antioxidants causes oxidative stress [22]. The increased ROS production induces the expression of genes that lead to dedifferentiation or senescence in chondrocytes [20]. Immunohistochemistry analysis of articular cartilage from mouse, human and non-human primates has shown increased nitrotyrosine, an oxidative damage marker in aged and osteoarthritic cartilage [23-25]. Oxidative stress accelerates the telomere shortening process, resulting in chondrocyte senescence and apoptosis of chondrocytes. Another mechanism of induction of chondrocyte senescence by oxidative stress is increased the level of expression of p53 and p21 and activation of p38 mitogen-activated protein kinase (MAPK) and PI3K/Akt signaling pathways which in turn triggers SASP [26]. Brandl and colleagues demonstrated that TRF1, TRF2, XRCC5, and Sirtuin-1 (SIRT1) expression is increased in human chondrocytes during the early passages following acute oxidative stress and decreases in the late passages. This demonstrates that these regulatory proteins deal with oxidative stress and protect DNA from damage in young chondrocytes. Due to reduced amounts of regulatory proteins in aged chondrocytes, they are more sensitive to oxidative stress, which leads to increased DNA damage and senescence [27]. Wnt family proteins are involved in the development of OA. It has been studied that the Wnt/β catenin pathway promotes chondrocyte senescence by increasing the expression of p53 and decreasing the expression of SIRT1 [28].

Apart from growth arrest, senescent cells show other vital features like secretion of proinflammatory cytokines, chemokines, and MMPS, also known as SASP. SASP is an important factor for the immune system to recognize senescent cells. As senescent cells accumulate with aging, it causes chronic inflammation, leading to the development of age-related diseases [29]. In OA cartilage, SASP contributes to the pro-inflammatory state and excessive synthesis of MMPs [30]. Freund and colleagues have studied the various SASP secreted by senescent cells and classified them according to their level of expression, which include high (>4 fold) to intermediate (2-4 fold) to small (<2 fold) [31]. Remarkably, OA tissue and synovial fluid showed all the SASP at high levels. Some of those high fold SASP involve Granulocyte macrophage-colony stimulating factor (GM-CSF), GROα, β, γ, insulin growth factor binding protein 7 (IGFBP-7), Interleukin-1 (IL-1α), IL-6, IL-7, IL-8, monocyte chemoattractant protein-1(MCP-1), MCP-2, macrophage inflammatory protein-1α (MIP1α), MMP-1, MMP-10, and MMP-3. The SASPs which are expressed at intermediates levels are intercellular adhesion molecule 1(ICAM-1), IL-1β, MCP-4, macrophage migration inhibitory factor (MIF), MMP-13, oncostatin M, regulated on activation, normal T cell expressed and secreted (RANTES), and tissue inhibitor of metalloproteinases 2 (TIMP-2). They have also been characterized as potential regulators in OA [30, 32-37]. Intercellular communication can trigger chondrocyte senescence in OA. Senescent chondrocytes secrete extracellular vesicles (EV), which negatively impact the healthy chondrocytes in the cartilage. It has been studied that the normal cells became senescent in human OA cartilage after receiving EV from senescent chondrocytes, and EV production was proportional to the number of senescent cells. This affects the matrix synthesis, which is mediated by the microRNAs derived from EVs [38, 39]. Expression of cellular communication network factor 1 (CCN1) and a gap junction channel protein connexin43 (Cx43) increases in senescent chondrocytes compared to normal chondrocytes [40, 41]. CCN1 stimulates senescence in chondrocytes by activating the p38 pathway and increasing SASP and MMP13 expression. Inhibiting CCN1 reduces inflammaging and maintains cartilage integrity [39, 41].

**Figure 1.**
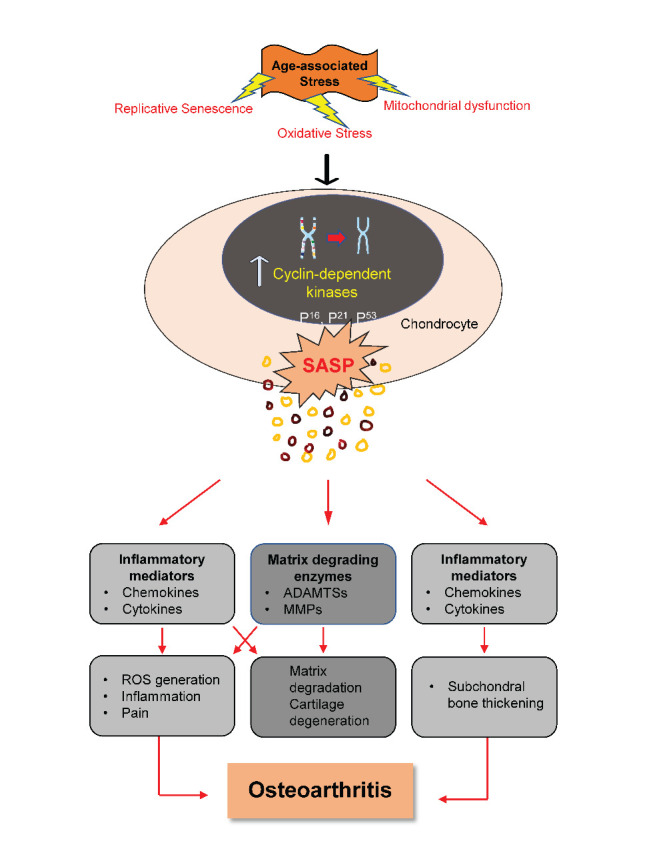
**Relationship between age-related changes, senescence, and development of OA.** Senescence is induced in chondrocytes by age-related stresses such as mitochondrial dysfunction, oxidative stress, and replicative senescence. These senescent cells express elevated levels of cyclin-dependent kinases such as p53 and secret SASP factors. The SASP factors trigger osteoarthritic changes in the synovial joint. SASP: senescence-associated secretory phenotype; ADAMTSs: A disintegrin and metalloproteinase with thrombospondin motifs; MMPs: Matrix metalloproteinases; IGFB: Insulin-like growth factor bindingproteins TFG- β: Transforming growth factor β; ROS- Reactive oxygen species

The reduction in chondrocyte proliferative and anabolic responses to growth factor stimulation is another hallmark of chondrocyte senescence. Stress-induced senescence reduces the ability of chondrocytes to synthesize type II collagen and aggrecan. In human chondrocytes, the increased expression of caveolin-1 protein induced by IL-1β can be inhibited by angiogenic growth factors (AGF) treatment. In the protection against chondrocyte senescence and cartilage aging, AGF may play a critical role [42]. The typical mitogenic response to multiple major growth factors, including TGF, bFGF, PDGF, and IGF-I, has declined with age [3]. This shows that the combined effect of aging, senescence and phenotypic changes in chondrocytes promotes cartilage degeneration, leading to OA progression. Figure 1 depicts how age-related stress impairs chondrocyte function in many ways and contributes to OA development.

### 2.2 Epigenetic regulation and aging

The expression of an age-dependent mechanism and epigenetics is an important mechanism in the development, aging, and age-related disorders [43]. Cells undergo extensive epigenetic modifications during the aging process. Epigenetics is a mechanism for regulating gene expression without altering the primary DNA sequence. Gene expression is regulated by epigenetic mechanisms such as DNA methylation, histone acetylation, and microRNA (miRNA) [44]. As epigenetic regulations are cell type-specific, disruption of a stable epigenetic state might lead to the development of several diseases, including OA. An increasing number of evidence show that an epigenetic mechanism alters the local transcriptional activity and expression of mRNA in articular cartilage chondrocytes to a significant extent [45, 46]. The epigenetic mechanism is critical for regulating specific cytokines, matrix proteins, and transcription factors gene expression [47].

**Figure 2.**
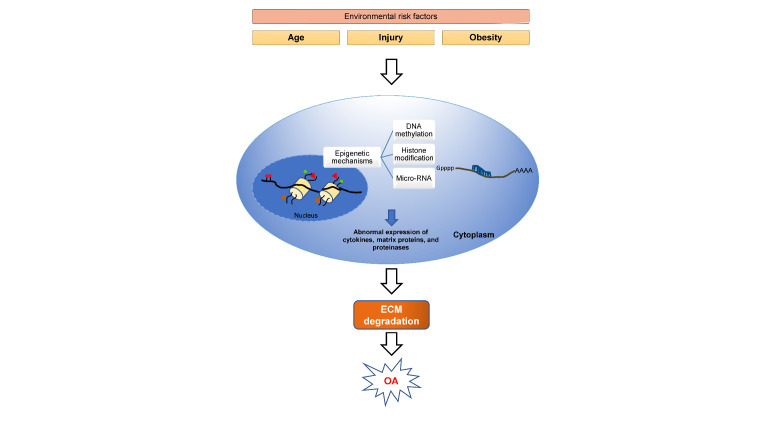
**Changes in epigenetic regulation and the development of OA.** Risk factors like age and obesity affect the stable epigenetic state of chondrocytes. DNA methylation and histone methylation changes occurred in the nucleus and miRNA expression in the cytoplasm. This affects the regulation of specific cytokines, matrix proteins, and gene expression and causes their abnormal expression, which affects the chondrocyte functions. These changes will lead to degeneration of cartilage and the pathogenesis of OA.

In chondrocyte differentiation and function, different growth factors like bone morphogenic proteins (BMPs), IGF-1, and TGF-β, play an essential role. BMP-7, also known as osteogenic protein-1 (OP-1), is important to matrix component synthesis. Alteration in the expression level of BMP-7 affects the homeostasis of the matrix. The OP-1 promoter's increasing methylation is one of the factors contributing to the decline in OP-1 mRNA expression with age [48, 49]. An SRY-Box transcription factor 9 (SOX9) transcription factor is involved in the differentiation of chondrocytes and cartilage formation. It has been demonstrated that, at embryonic and newborn stages of mice, SOX9 expression is highest, but it decreases significantly in completely developed joints. This age-dependent SOX9 expression is regulated by DNA methylation and histone methylation in mouse articular cartilage [50]. In mouse cartilage, postnatal inactivation of SOX9 showed a decrease in proteoglycan content but did not show any sign of OA development by the age of 18 months; however, in humans, reduction in SOX9 expression leads to the development of OA [51, 52]. In human chondrocytes, miRNA-145 inhibits the expression of SOX9. The expression of collagen type II alpha one chain (COL2A1) and aggrecan is dramatically reduced when miRNA-145 levels increase, whereas the expression of matrix metalloproteinase is significantly increased. (MMP-13) [53]. Another transcription factor, Nfat1 (NFAT1/NFATc2), is a nuclear factor of activated T cells (NFAT) family member that plays a critical role in maintaining the articular cartilage structure in adult mice. Nfat-deficient mice do not affect skeletal development. Still, it affects the chondrocyte function and exhibits OA-like changes such as increased expression of proinflammatory cytokines, the imbalance between anabolic and catabolic, and loss of cartilage repair abilities [54]. It has been proven that while chondrocyte NFAT1 expression is low at the embryonic stage, it is high in adult chondrocytes in wild-type mice. Histone methylation controls this age-dependent expression of NFAT [55]. Figure 2 depicts the impact of risk factors such as aging in causing significant epigenetic changes, which eventually contribute to the development of OA.

### 2.3 Autophagy and aging

Autophagy is a protective mechanism of cells to maintain cellular integrity. It is a process to degrade the cells' damaged and dysfunctional cell organelles, proteins, and other macromolecules. Autophagy enhances the function of the articular chondrocyte. Moreover, autophagy dysfunction is linked with the pathogenesis and increased severity of OA. Autophagy decline or abnormalities are related to accelerated aging-related alterations; however, autophagy stimulation may have anti-aging effects [56-59]. It has been studied that aging cartilage from humans and mice shows the reduced the expression of autophagy proteins like uncoordinated-51-like protein kinase (ULK1), Beclin1, and light chain 3 (LC3) expression [57]. In a mouse model, reduced autophagy activity is linked to the mechanisms of aging-related OA, which affects cell and tissue homeostasis and leads to the development of joint structural defects [60].

Around 30 autophagy-related genes (ATGs) are involved in the process of autophagy. Autophagy is regulated by the mammalian target of rapamycin complex 1 (mTORC-1). It inhibits the ULK1 complex by acting as a negative regulator. Increased mTOR expression is linked to increased chondrocyte apoptosis and decreased expression of key autophagy genes in OA cartilage [61]. In C57Bl/6 OA mice, rapamycin treatment which inhibits mTORC-1 and activates autophagy, retains cartilage cellularity and lowers IL-1β expression in articular cartilage [62]. The damaged or dysfunctional cellular components are enclosed in autophagosomes, a double-membrane vesicle during autophagy. The autophagosome formation is regulated by ATG proteins, including ATG-12, ATG-5, and LC3. During autophagy, LC3-I is converted to LC3-II, an important autophagy marker. SIRT1, an NAD+ dependent histone deacetylase of class III, regulates age-related physiological functions such as stress responses. It has been shown that in human chondrocytes, SIRT1 regulates autophagy via interacting with ATG-7 [63]. SIRT1 expression levels in human chondrocytes decline with age and decreases cartilage integrity with aging. As SIRT1 causes deacetylation of autophagy proteins and increases autophagy, this shows that it is essential to maintain autophagy in chondrocyte and cartilage integrity; therefore, targeting SIRT1 might be important in the prevention of OA [64]. Xu and colleagues have demonstrated that Sirtuin-3 (SIRT3), a deacetylase, activates autophagy by inhibiting PI3K/Akt/mTOR and plays a critical role in protecting against OA pathophysiology [59].

Autophagosome fuses with lysosome where the degradation occurs [57, 65]. Lysosomes play an important role in the degradation of cellular waste and in the process of autophagy. Impaired function of the lysosome is associated with an increase in the chondrocyte apoptosis via release of the Cytochrome c, a proapoptotic protein which induces apoptosis by the Caspase3/7 dependent pathway. Autophagy is continuously activated in cartilage; however, the level of constitutive autophagy in aged mice decreases, and the size and number of autophagosomes are reduced [66]. The autophagy proteins like ATG-5 and LC3 expression levels are reduced in aged mice [67]. While OA cartilage also shows a reduction in levels of autophagy genes like ATG3, ATG12, ULK1, beclin 1, and γ-aminobutyric acid receptor-associated protein-like one and shows an increase in apoptosis [57]. Autophagy has a protective role in cells however, the complete understanding of the mechanism of autophagy and lysosome dysfunction in aged and osteoarthritic cartilage has great therapeutic potential for the treatment of OA because of its ability to target the aging process of chondrocytes.

### 2.4 Mitochondrial dysfunction and aging

Mitochondria play a critical role in the aging process. Mitochondria are subcellular organelle mainly involved in energy production by oxidative phosphorylation. But the function of mitochondria declines with age. The mitochondrial dysfunction is viewed as a “hallmark of aging.” The mitochondrial dysfunction also promotes the development of age-related diseases like OA [68]. It has been studied that mitochondrial dysfunction results in increased production of ROS, which increases oxidative stress, causes a reduction in ATP production, decreases matrix synthesis ability, and increased apoptosis in osteoarthritic chondrocytes [68, 69]. Mitochondrial dysfunction in aged and OA cartilage activates JNK-MAPK/cFos/AP1 pathway via ROS and triggers the production of matrix-degrading enzymes and the expression of proinflammatory genes [70]. Increased ROS generation activates the MAPK and MAPK/ERK signaling pathways, limiting the formation of extracellular matrix (ECM) components such as collagen II and glycosaminoglycans while promoting type I collagen production [71]. ROS overexpression also damages mitochondrial DNA (mtDNA) and impairs its repair capacity [72].

Superoxide dismutase (SOD) catalyzes the conversion of superoxide to hydrogen superoxide and molecular oxygen, therefore maintaining redox homeostasis. Superoxide dismutase 1, 2, and 3 levels were reduced in osteoarthritic cartilage, but SOD2 knockout mice demonstrated increased cartilage degradation with age [73, 74]. It has been shown that in OA chondrocyte Aurora kinase A (AURKA) binds to SOD2 and causes its lysine-48 (K48) ubiquitination mediated degradation, resulting in mitochondrial dysfunction [75]. Fu et al. have shown that, even though there is an increased expression of superoxide dismutase in mitochondria in aged mice, its specific activity decreases with age due to increased posttranslational lysine acetylation. In cartilage Sirtuin-3 (SIRT3), mitochondrial deacetylase levels decrease with age, driving a decline in the SOD-2 activity. Incubation of aged chondrocyte homogenates with SIRT3 and NAD^+^ showed an increased level of SOD2 activity [76, 77]. Maintaining SIRT3 levels in aging cartilage could be a potential therapeutic strategy to retain SOD-2 levels in aging cartilage and alleviate age-related mitochondrial dysfunction [76, 78].

### 2.5 Growth factor response and aging

Various growth factors play a key role in regulating different signaling pathways in articular cartilage. They are important for cell growth, division, and differentiation, thus influencing cartilage development and function. As chondrocytes age, their responsiveness to growth hormones like insulin-like growth factor-1 (IGF-1) and transforming growth factor - β (TGF - β) decreases, which results in impairs chondrocyte ability to repair. IGF-1 is a key factor in articular cartilage that promotes proteoglycan production and is required for cartilage integrity [79]. IGF-1 also promotes chondrocyte proliferation while inhibiting the terminal differentiation of chondrocytes. It has been shown that an increase in IGF binding proteins could result in chondrocytes’ ability to respond to IGF-1. When compared between young and old rats, there is a significant decrease in proteoglycan synthesis [80]. Another study indicates that the age-related reduction in the responsiveness of chondrocytes to IGF-1 could be due to the aged cells’ inability to adequately transduce the signal generated from the IGF-1 receptor [81]. IGF-1 binding to the IGF-1 receptor activates various signaling pathways like Shc/Grb2/Sos/Ras/Raf/MEK/ERK/MAPK pathways. The phosphorylation of insulin receptor substrate 1 (IRS-1) and insulin receptor substrate 2 (IRS-2) occurs when IGF-1 binds to its receptor called IGF-1 receptor, receptor, which further stimulates various signaling pathways, such as the PI3K (phosphoinositide 3-kinase) cascade and ERK (extracellular-signal-regulated kinase). PI3K then leads to the activation of AKT [82]. Activation of Akt signaling by IGF-1 promotes proteoglycan and collagen type II synthesis, while activation of ERK has inhibitory effects [83]. It has been studied that; aged chondrocytes show a decline in proteoglycan synthesis compared to young chondrocytes. The level of Akt phosphorylation is lower in aged chondrocytes than in young chondrocytes [83]. During the development of OA, decreasing levels of IGF-1 are also important in shifting the balance towards catabolic metabolism. Excessive ROS inhibits the IRS-1-PI-3 kinase-Akt signaling pathway. This signaling pathway is important for promoting matrix production. Simultaneously, ROS activates the ERK/MAP kinase and decreases the production of aggrecan, type II collagen, and Sox-9 [84, 85]. Through oxidized low-density lipoprotein (LDL, extracellular ROS may also inhibit the Akt pathway. Stress-induced chondrocyte senescence is triggered by oxidized LDL binding to its receptor, lectin-like ox-LDL receptor 1 (LOX-1), which is associated with a lower Akt phosphorylation than after IGF-1 stimulation [86, 87].

TGF- β is an essential anabolic growth factor in chondrocyte differentiation and plays a vital role in maintaining healthy cartilage. TGF- β acts via two different receptors and generates two different responses. TGF-β signaling via activin receptor-like kinase-5 (ALK5) promotes the synthesis of matrix components like aggrecan and collagen, while signaling via activin receptor-like kinase - 1 (ALK1) induces the expression of MMP-13 [88]. The response of aged chondrocytes and chondrocytes from OA cartilage to TGF-β is reduced, and even the expression of the ratio of receptor ALK1 to ALK5 changes in chondrocytes from aged and OA cartilage. Increased catabolic activity in chondrocytes relative to anabolic activity is the outcome of these alterations in receptor expression, and this imbalance leads to cartilage deterioration [89]. A better understanding of age-associated changes in growth factor response and changes in their receptor interaction would provide an innovative approach for the development of new strategies for OA treatment.

### 2.6 Chondrocyte metabolism and aging

Articular cartilage is an avascular tissue. Due to a lack of direct oxygen supply, chondrocyte metabolism is constrained by a low degree of anaerobic glycolysis generated by the slow oxygen diffusion and nutrients through the synovial fluid. Under normal oxygen tension, chondrocytes show aerobic glycolysis (Warburg effect) and anaerobic glycolysis [90]. OA cartilage shows a higher rate of anaerobic glycolysis. Glucose transporter (GLUT) 1, a membrane-embedded protein important for glucose transport in chondrocytes, plays a vital role in chondrogenesis [91]. In a hypoxic environment and in response to pro-inflammatory cytokines, GLUT1 expression is increased considerably in chondrocytes [74, 92]. Increased glucose uptake and excessive Advanced Glycation End-products (AGE) are caused by constant, enhanced GLUT1 expression, which can damage cartilage [93]. Pyruvate kinase M2 (PKM2), an isoenzyme of pyruvate kinase, is significantly expressed in OA cartilage chondrocytes; however, PKM2 knockdown reduces COL2A1 and SOX9 expression and promotes chondrocyte death, implying that PKM2 may play a role in the progression of OA [94]. The tricarboxylic acid cycle is the key metabolic pathway in cells, generating more energy than glycolysis. However, due to the increased rate of anaerobic glycolysis in OA chondrocytes, fewer pyruvate molecules are decarboxylated to form acetylated-CoA for the tricarboxylic acid cycle [95, 96].

Cholesterol levels have a critical role in the development of OA. Mice fed with a cholesterol-rich diet and mice without the low-density lipoprotein receptor (LDLr-/-) demonstrated osteophyte development [97]. Intracellular accumulation of cholesterol promotes the severity of OA. Hedgehog (Hh) signaling alters cholesterol accumulation in chondrocytes by regulating cholesterol homeostasis genes. Reduced cholesterol accumulation via HH signaling reduces cartilage degeneration [98].

## 3. Age-related changes in the ECM composition and characteristics

Articular cartilage (AC) is hyaline cartilage composed of extracellular matrix and chondrocytes. ECM is the complex network of macromolecules, around 95% of the cartilage consist of ECM and chondrocytes. Chondrocytes are responsible for ECM synthesis, and it is composed of collagen, glycosaminoglycans (GAGs), proteoglycans, glycoproteins, minerals, fibrous proteins, and 70 - 80% water. The prime function of ECM is to provide structural support to the tissue and maintain tissue stability [99]. ECM of cartilage directly impacts the tissue's response to mechanical strain, which is critical for maintaining its composition and functionality. The age-related changes increase the susceptibility to cartilage damage, which may lead to OA development. With aging, there is a gradual loss of the cartilage matrix. The loss of chondrocytes, reduced activity of chondrocytes to growth stimuli, or a decrease in water content could all contribute to cartilage thinning [23, 27].

### 3.1 Collagen II

Collagen type II is predominant in mature articular cartilage. It forms fibrils with proteoglycan and collagen IX and XI. Other collagen types like collagen I, IV, V, VI, IX, and XI are present in less proportion. These collagens support the collagen II fibrils network, and these fibrils provide tensile strength to the cartilage [99]. Numerous collagenases' expression and activity increase with age, resulting in fibrillation and cartilage disintegration, which affect the ECM's stability [100, 101]. Collagen undergoes structural changes with age, resulting in a decrease in the elasticity of the collagen fibers and ultimately altering the collagen's biomechanical properties [99]. With increasing age, the thickness of collagen fiber increases. This is due to non-enzymatic modifications in collagen resulting in the formation of AGE, and it starts to accumulate with increasing age [102]. Accumulation of AGE products makes cartilage stiffer and brittle with advancing age [103]. AGE accumulates in aggrecan, but it accumulates more with collagen, causes excessive crosslinking with collagen, and changes the biomechanical properties of cartilage by increasing the stiffness of the matrix and making cartilage more vulnerable to mechanical failure [99, 103, 104]. Kim and colleagues demonstrated that increasing AGE and the collagen crosslinking enzyme lysyl oxidase increases cartilage stiffness in a surgically induced mouse model [105]. AGEs interact with cell surface receptors called Receptor for Advanced Glycation End-products (RAGE) present on the articular chondrocyte. It has been studied that the levels of RAGE increase during aging and OA. AGE binding activates the RAGE signaling and stimulates the production of MMP-13 [106]. Additionally, AGE accumulations increase inflammation in the cartilage by increasing TNFα as well as the inflammatory mediators like prostaglandin E2 and nitric oxide. It also affects the type II collagen expression while MMPs, a disintegrin, and metalloproteinase with thrombospondin motifs expression are increased (ADAMTS) [107, 108]. It induces apoptosis by activating the NF-кB pathway, leading to further damage to the tissue [104, 109, 110].

**Figure 3.**
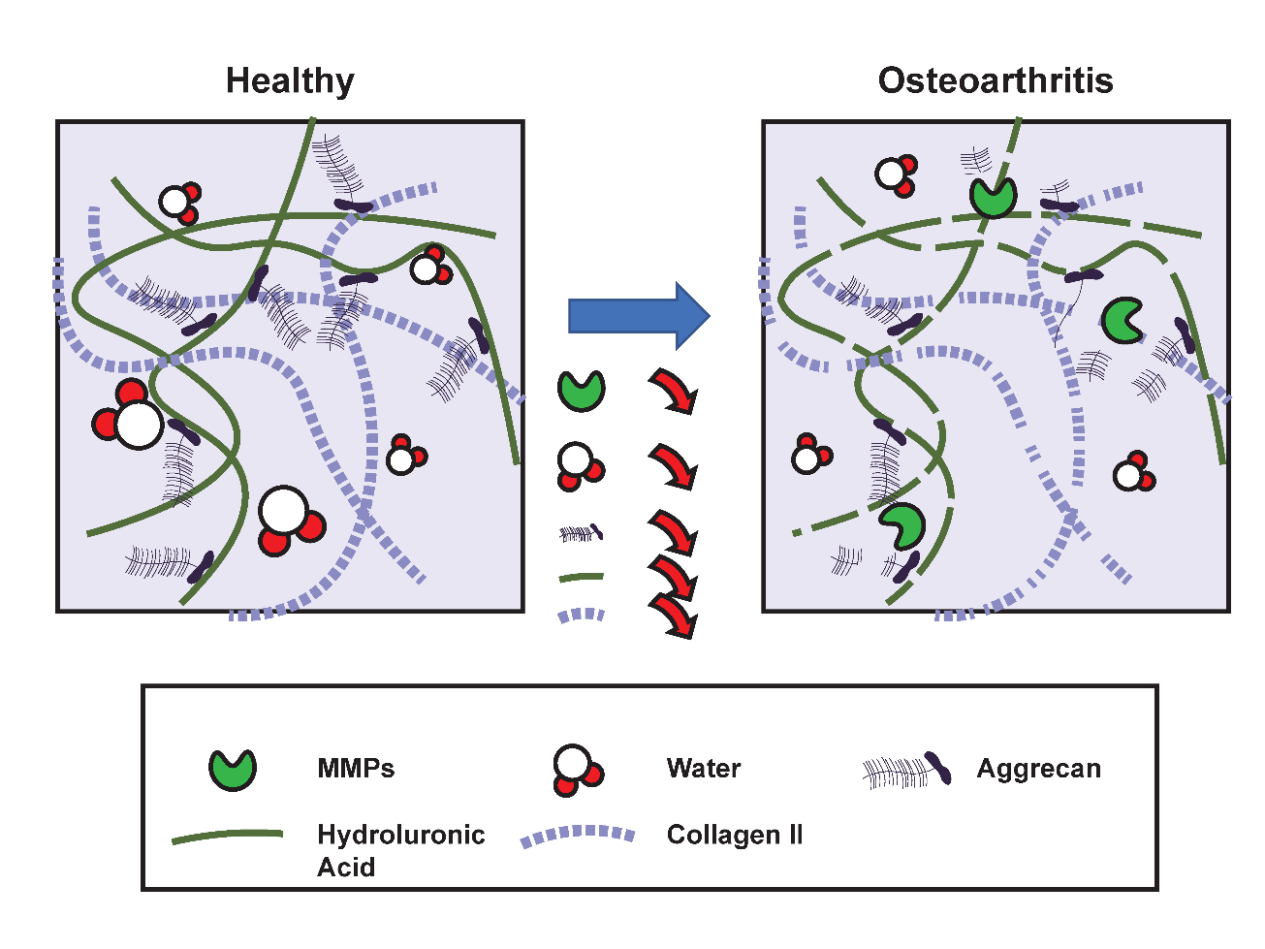
**Changes in ECM structure in healthy and OA cartilage.** Under normal physiological conditions, a healthy cartilage structure is maintained by an underlying network of collagen II and aggrecan aggregates with a hyaluronic acid backbone. However, osteoarthritic cartilage exhibits elevated MMPs, and reduced aggrecan molecule size. MMPs: Matrix metalloproteinases

### 3.2 Proteoglycans

Proteoglycans (PG) are highly glycosylated protein cores attached covalently to GAG chains [111]. PGs are highly negatively charged macromolecules that represent 10 to 15% of the wet weight, attract water, and salts in the cartilage, and give hydrophilic properties to cartilage. The ECM includes various proteoglycans that include aggrecan, decorin, biglycan, and fibromodulin [112]. Aggrecan is negatively charged, highly abundant proteoglycans and occurs in the form of aggregates. Aggrecan interacts with GAG and hyaluronan and provides osmotic properties to the cartilage, essential to resist the compressive load [113]. With increasing age, the number and size of proteoglycan aggregate decreases, reducing cartilage water content [114, 115]. An increase in proteolytic activity of aggrecanases leads to the release of aggrecan in the synovial fluid. The concentration of aggrecan fragments "ARGS" in the serum is age-related and increases in OA patients [116, 117]. Proteoglycan plays an important role in the transport of solute and nutrients transport. Age-related changes in cartilage affect the diffusion of nutrients in cartilage [118]. Deposition of calcium-containing crystals, primarily calcium pyrophosphate and basic calcium phosphate (BCP), is a major alteration in aged cartilage. Cartilage calcification due to BCP is associated with chondrocyte hypertrophy and the severity of OA [119, 120]. This cartilage calcification in the synovial joint is primarily due to aging rather than OA, and it serves as a precursor to increased fibrillation and OA[121]. Figure 3 shows the primary changes in ECM that occur with age.

Apart from structural support, ECM is also important for cell to cell signaling and matrix to cell signaling. Matrix to cell signaling plays an important role in the process of mechanotransduction [122]. Integrins are transmembrane proteins that act as a connecting link between ECM and cell cytoskeleton. Integrin enables mechanical signal transmission from ECM to cell and generates the intracellular response. The cytoskeleton acts as a mediator between chondrocytes and ECM interaction and senses the mechanical stimuli [123]. Nofal and Knudson have shown that disruption in the cytoskeletal structure of chondrocytes uncouples it from the extracellular matrix, resulting in altered metabolism and deleterious changes in a matrix structure [124].

## 4. Conclusion and perspective

The prevalence of OA is increasing with age. Aging affects the musculoskeletal system at a molecular and functional level. The regenerative potential of bone and cartilage is affected by cell renewal and matrix alterations. However, the molecular pathways that link aging and OA are not fully understood. We have summarised current information about the physiological and pathological alterations at the cellular and tissue levels that can be associated with joint aging and contribute to the development of OA.

In this review, we have summarised the role of aging in the development of OA. While aging and OA are closely linked, aging does not always result in the formation of OA. However, age-related alterations in chondrocytes and the ECM increase the joint's susceptibility to OA development. The cumulative effect of risk factors like senescence, altered epigenetics, mitochondrial dysfunction, changes in cell metabolism, and growth factor response affects chondrocyte homeostasis. These changes ultimately affect the homeostasis of ECM and lead to cartilage degradation. All the changes are the prime risk factors for the development of OA.

A deeper understanding of the aging mechanism may enable the creation of novel strategies for delaying these changes and for the development of therapeutics that aid in the slow progression of age-related OA development. Understanding age-related changes in the synovial joint aids in the development of specialized therapies that contribute to disease prevention. Numerous studies have demonstrated that cell-based therapies such as stem cell therapy or autologous chondrocyte implantation may be effective in reducing the severity of OA. Cell-free therapy is another current major therapy that shows promising outcomes in the treatment of OA. Numerous studies have demonstrated that extracellular vesicles are a promising therapy option for OA. Treatment options like use of extracellular vehicles and early detection of OA by targeting biomarkers need to develop to prevent the progression of OA.
